# Frequency Dependence of Intrinsic Stress and Birefringence Tensor of Bead/Spring Model of Polymer Solutions

**DOI:** 10.6028/jres.081A.012

**Published:** 1977-02-01

**Authors:** A. Peterlin, J. T. Fong

**Affiliations:** Institute for Materials Research, National Bureau of Standards, Washington, D.C. 20234; Institute for Basic Standards, National Bureau of Standards, Washington, D.C. 20234

**Keywords:** Bead-spring model, eigenvalues, frequency response, intrinsic optical tensor, intrinsic stress tensor, polymer solution

## Abstract

The recently obtained complete solution of the simultaneous diagonalization of matrices **H A** and **H** in the hydrodynamic diffusion equation has basically changed the diagonal values *v_p_* of the symmetric matrix **H** of hydrodynamic interaction between all the beads of the elastic random coil model of the isolated macromolecule in solution. Since these values enter explicitly the expressions for the intrinsic stress and refractive index tensor in an alternating flow field if based on the concept of internal viscosity of the model one had to recalculate all values obtained formerly by using the then generally accepted erroneous set of *v_p_* data. The new *v_p_* equal unity independent of *p* while the old values were larger than 1 for small *p* and smaller for large *p.* Hence their too large contributions in the former range are partially compensated by their too small contributions in the latter region. As a consequence in the whole range investigated, between 3 and 300 chain links, the differences in rheological and rheooptical effects are relatively small, up to a factor of 2, although at higher link number the differences tend to grow with the logarithm of this number.

## 1. Introduction

The correct simultaneous diagonalization [1]^1^ of **H A** and **H** matrices in Zimm’s hydrodynamic equation [2] for the ideally flexible necklace model of randomly coiled isolated linear macromolecule in laminar flow makes possible a more adequate calculation of intrinsic stress tensor in all those cases where the coil is not yet noticeably deformed by the flow. Such a zero gradient case includes the frequency dependence of viscosity [2], shear modulus, shear birefringence, normal stress difference, and acoustic birefringence but not the gradient dependence of these effects.

The main change introduced by the new solution as compared with the older incomplete solutions [3–8] is not in the eigenvalues λ*_p_* of **H A**

$$Q^{-1}\;H\;A\;Q=\Lambda$$ (1)

which were already calculated correctly in recent papers [6–8] and even tabulated for *Z* between 1 and 15 and h* = (3/*π*)^1/2^ *a_h_/b*_0_ = .01, .1, .2, .3, [6] and for *Z* = 250, h* = .3 and *Z* = 300, h* = 0.4 [1]. Here *a_h_* is the hydrodynamic radius of the bead, *Z* + 1 is the number of beads, *Z* is the number and *b*_0_ the root mean square length of the links. The difference is in the diagonal elements *v_p_* of the tensor

$$Q^{-1\;}H\;Q^{-1T}=N=1$$ (2)

which turns out to be the unity tensor with all *v_p_* = 1. This makes the diagonal elements of

$$M=Q^{T\;}A\;Q=\Lambda /N=\Lambda$$ (3)

agree with those of **Λ**, i.e. λ*_p_* = λ*_p_.* Here **Q** is the transformation matrix of the original 3(*Z* + 1) dimensional vector ***r*** of bead coordinates to that of dimensionless normal coordinates ***u***

$$r=b_{0}\;Q\;u$$ (4)

and **Q***^T^* its transpose.

Equation 3 completely differs from the original estimate [3] that in first approximation the diagonal elements of the matrix **M** equal the eigenvalues *λ_pR_* of the Rouse model with vanishing hydrodynamic interaction, i.e.

$$\mu_{pZ}^{\left(1\right)}=\lambda_{pR}=4\;\sin^{2}\left[p\pi /2\left(Z+1\right)\right]$$ (5)

and hence

$$v_{pZ}^{\left(1\right)}=\lambda_{pZ}/\lambda_{pR}.$$ (6)

The subscripts *Z* and *R* refer to Zimm and Rouse [9] model, respectively. Such an estimate was based [3] on the supposition that the transformation matrix **Q** changes so little by the introduction of hydrodynamic interaction that **M** remains practically the same as in the free draining case, i.e. **M =** *λ_R_*.

**Figure 1:**
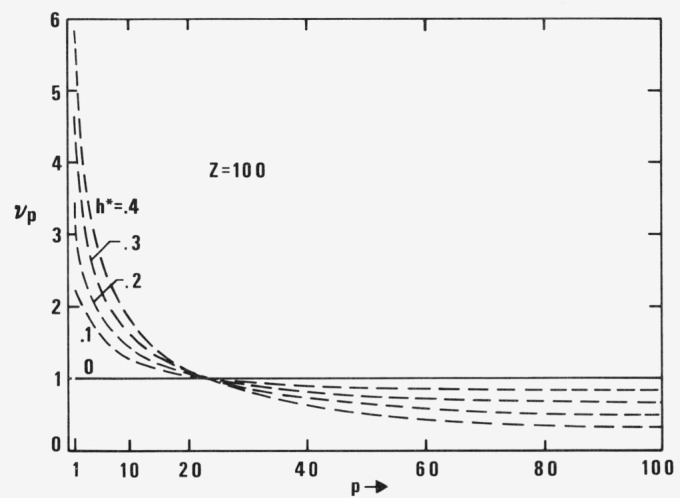
*Diagonal elements v_p_* (*new theory) and* $v_{p}^{\left(1\right)}=\lambda_{\text{pZ}}/\lambda_{\text{pR}}$ (*old theory, broken lines) for Z* = 100 and h* = 0, .1, .2, .3, .4. For h* = 0 (Rouse) the values of both theories coincide.

The diagonal elements *v_p_* for *Z* = 100 and h* = 0 (Rouse), .1, .2, .3, and .4 (Zimm) are plotted in figure 1 and the eigenvalues λ*_p_* collected in table 1. The old values of $v_{p}^{\left(1\right)}=\lambda_{\text{pZ}}/\lambda_{\text{pR}}$ are connected with a broken line. They are partially situated below and partially above *v_p_* = 1. This reduces the differences between the old and new values in quantities dependent on *v_p_.* They show up in the excess stress tensor as soon as internal viscosity, defined by the frictional parameter *φ*, is explicitly considered. The very large old values *v_p_* for small *p* do not matter very much because in all formulae they are multiplied by very small values *φ_p_ = pφ*/*Z.* Since the differences between old and new *v_p_* increase with h*, in that which follows, the comparison of calculated effects will be mainly done for h* = .4, i.e., for a very large hydrodynamic interaction. The differences are smaller for smaller h* and of course disappear for the free draining coil with h* = 0 where λ*_pZ_* = λ*_pR_*.

The new values *μ_p_* and *v_p_* do not enter the conventional expressions for the intrinsic stress or birefringence tensor of perfectly flexible necklace model so that no changes occur in these quantities. The situation is completely different if one considers the effects of internal viscosity which depend on *v_p_* [10–26]. They will be most conspicuous in the values of viscosity corresponding to high frequency flow field and in the phase angle between the stress and strain rate or between the birefringence and strain rate.

The interaction tensor **H** depends on the inverse intrabead distances 1 */r_jk_* which makes the hydrodynamic diffusion equation intrinsically non-linear. By replacing 1 */r_ik_* with its average value ⟨*1/r_jk_*⟩ the tensor **H** becomes a constant which makes the diffusion equation linear and hence allows the introduction of normal coordinates according to eqs (1) to (4). Such a procedure eliminates the possibility of any realistic consideration of the gradient dependence of any rheological or rheooptical effect because it does not take into account the change of shape of the random coil in flow which expands the molecule and hence increases the interbead distances *r_jk_.* Note also that by preaveraging over all angles between the velocity and the interbead vector the formulation of **H** as function of 1 */r_jk_* completely evades the consideration of anisotropy of hydrodynamic interaction which by itself yields a gradient dependence of intrinsic viscosity [27] large enough for explaining experimental data.

The general toleration of such a profound modification of hydrodynamic interaction by the replacement of 1 */r_jk_* with its average ⟨1 */r_jk_*⟩ makes hard to understand the almost general objections to the introduction of internal viscosity as a resistance of the necklace model against the deformational component of the normal eigenmodes [28–30]. If one accepts the rather questionable linearization of the hydrodynamic diffusion equation one has to accept also the next step, i.e., the concept of internal viscosity based on this linearity and its introduction in such a manner that the mathematical treatment remains as simple as possible.

In that which follows the results of the new theory will be compared with those of the old one for h* = .4 and *Z* = 100 in the whole frequency range and the dependence of the limiting values for *ω = ∞* on *Z* in the range between *Z* = 3 and 300 and on h* in the range between .1 and .4. In all cases the ratio between the internal viscosity coefficient *φ* and the frictional coefficient of the bead *f* = *6πa_h_η_s_* will be assumed constant, *φ*/*f* = 2. Here *η_s_* is viscosity of the solvent. The subscript *s* applies to the properties of the solvent. The corresponding non-subscripted quantities relate to solution.

## 2. Internal Viscosity

The concept of internal viscosity was introduced in order to express the inability of the randomly coiled polymer molecule to change rapidly its shape [31, 32, 10, 11]. Such changes occur during the rotation of the macromolecule in a flow with a rotational component, e.g., the laminar flow with transverse gradient, when the individual segments are alternatively passing from the direction of compression to the direction of extension and vice versa. The directions of maximum compression and extension of the volume element are in the flow plane perpendicular to each other. The rapidity of change is given by the transverse gradient which equals twice the angular velocity of the ideally flexible coil which rotates with the volume element. The other case is the oscillating flow field where the oscillation frequency determines the rapidity of change from compression to extension and vice versa.

In the limiting cases of zero gradient and zero frequency the deformation inability of the macromolecule does not play any role. All the changes occur so slowly that the effects are identical for completely rigid and ideally flexible coils if only their conformational distributions agree with each other. With increasing gradient and/or frequency, however, the time effects are playing a gradually increasing role. They are maximum in the second Newtonian regime corresponding to $\dot{\gamma }\to \infty$ or *ω* →∞.

The rigidity of the macromolecule can be assigned to different properties of the chain. It can be caused by the energy barrier separating the gauche and trans conformations which makes any conformational change more time consuming than in the case of perfectly soft model [10]. Since the height of the energy barrier is independent of the viscosity of the solvent its relative effect as measured by the ratio of internal frictional resistance *φ* caused by the barrier and the frictional resistance *f* of the segment decreases with the viscosity of the solvent. The macromolecule acts as very rigid in a low viscosity liquid, e.g., acetone with *η_s_* = .322cPoise = 32.2 mNs/m^3^, and as very flexible in a high viscosity solvent, e.g., Aroclor with *η_s_* up to 100 Poise (= 1 kNs/m^3^) and higher.

Another cause of slow molecular response to the rapidly changing flow field resides with the conformational restraints of the chain which permit only an interchange of gauche and trans conformations [20, 23, 24]. With almost rigid length of valency bonds this means that most changes of length and position of any chain segment require a much larger segment displacement than formulated in the ideally flexible necklace model which does not consider any inherent limitation of bead motion. Generally an axial displacement of the segment requires also some lateral displacement and vice versa. As a consequence the resistance of beads to position change is larger than assumed on the basis of hydrodynamic radius *a_h_.* The ratio of the so obtained coefficient *φ* to *f* is independent of solvent viscosity because *φ* and *f* are both proportional to *η_s_.* Their ratio just measures the ratio of true displacement to the minimum displacement explicitely considered in the diffusion equation. It seems to be close to 2 for vinyl polymers

The effect of internal viscosity is formulated in the system of normal modes [10]. For the *p*th normal mode of deformation one has a resistance coefficient *φ p*/*Z.* Such a choice is reasonable for both origins of internal viscosity as just discussed. In the first case one can argue that the changes to comply with any deformational mode are linearly increasing with the number of chain atoms between subsequent nodes, i.e., with *Z/p*, because a conformational change takes place with equal probability at any of these chain atoms. This makes the resistance increase with *p/Z.* In the second case the displacement at lower modes can be achieved in many ways so that the actual lengthening of displacement path is much less noticeable than at higher modes where the conformational restrictions are soon becoming of overwhelming importance.

One may argue that the whole concept of internal viscosity can be discarded because it is not based on some strictly fundamental analysis of chain dynamics. It was indeed introduced in a rather pragmatic manner which also permitted an easy mathematical treatment [10]. But it turns out that all more detailed treatments of Brownian motion of beads or of correlation between the motion of two or more beads [33–35] lead to some, often hidden, statement of molecular rigidity which is needed for the results of such a study to reproduce the characteristic rheological features of polymer systems, e.g., the non-vanishing limiting intrinsic viscosity at very high frequency [36–51]. Such a state of affair seems more to support than to refute the concept of internal viscosity in spite of its more pragmatic than fundamental way of introduction.

## 3. The Distribution Function of the Beads

The continuity equation of the ideally flexible necklace in laminar flow which determines the distribution function *ψ*(**r**_0_, ***r***_1_ ⋯ ***r****_z_*) reads

$$\begin{array}{l} \nabla_{r}^{T}\left[v\psi -\left(3D_{0}/b_{0}^{2}\right)H\;A\;r\;\psi -D_{0}\;H\;\nabla_{r}\;\psi \right]\\ \;=-\partial \psi /\partial t. \end{array}$$ (7)

Here *D_0_ = kT*/*f* is the translational diffusion coefficient of the bead. By introduction of normal coordinates, eq (4) one transforms eq (7) into a system of *Z* + 1 partial differential equations

$$\begin{array}{l} \nabla_{p}\left[v_{p}\;\psi_{p}-\left(3D_{0}/b_{0}^{2}\right)\lambda_{p}u_{p}-\left(D_{0}/b_{0}^{2}\right)v_{p}\;\nabla_{p}\;\psi_{p}\right]\\ \;=-\partial \psi_{p}/\partial t \end{array}$$ (8)

each depending only on the coordinates of the *p*th eigenmode. Note that ***v****_p_* in normal coordinates has the dimension *s*^−1^. The distribution function of the coil is the product of all *ψ_p_*

$$\psi \left(u,t\right)=\psi_{1}\left(u_{l},t\right)\cdots \psi_{Z}\left(u_{Z},t\right).$$ (9)

The functions *ψ_p_* depend on the kind of flow field *v*. The 0th mode does not show up in *ψ* because it represents a uniform translation of the whole necklace which does not affect *ψ.*

The introduction of internal viscosity adds a viscous type resistance coefficient (*φ_p_ = pφ/Z* opposing the *p*th eigenmode of the true deformation rate of the coil. This rate is obtained by subraction of pure rotational velocity Ω × ***u****_p_* from the total deformation rate *∂****u****_p_/∂t.* This yields an internal viscosity force [10, 11]

$$F_{ip}=-\left(p\varphi /Z\right)\left(\partial u_{p}/\partial t-\Omega \times u_{p}\right)b_{0}.$$ (10)

If one introduces this force in the *p*th diffusion equation eq (8)) one obtains after some rearrangements

$${\left(1+v_{p}\varphi_{p}/f\right)}^{-1}\nabla_{p}\left\{\left[v_{p}-\left(3D_{0}/b_{0}^{2}\right)\lambda_{p}u_{p}+\left(v_{p}\varphi_{p}/f\right)\Omega \times u_{p}-\left(D_{0}/b_{0}^{2}\right)v_{p}\nabla_{p}\right]\psi_{p}\right\}=-\partial \psi /\partial t.$$ (11)

The distribution function *ψ_p_* depends on the kind of flow and on the angular velocity vertor Ω.

In a jet or plane flow with longitudinal gradient without a rotational flow component one has Ω = 0. In a flow with transverse gradient, $v=\dot{\gamma }\left(y,o,o\right)$, the angular velocity equals − *γ*/2

$$\Omega =\left(0,0,-\dot{\gamma }/2\right)$$ (12)

for relatively soft molecules which rotate in phase with the volume element. This is the case with practically all conventional macromolecules if the degree of polymerization is so high that a truly random coil is formed. Very short chains, chains with a great many double bonds, ladder type and multiple strand molecules, however, are more rigid and tend to rotate with a non-uniform angular velocity which depends on orientation of the molecule. It is different from that of he volume element. Assymptotically, at very high rigidity and fully extended shape of the macromolecule, it approaches that of rigid bodies, e.g., rods or ellipsoides. In that which follows only the case of practically undeformed relatively soft coils with ( $\Omega =-\dot{\gamma }/2$ will be considered.

In the steady state flow with transverse gradient the *p*th eigenmode distribution function of the soft necklace reads

$$\begin{array}{l} \psi_{p}\left(\xi_{p},\eta_{p},\xi_{p}\right)={\left(\frac{\mu_{p}}{\pi }\right)}^{3/2}{\left(\frac{1+\beta_{p}^{\prime }{\;}^{2}-\beta_{p}^{2}}{1+\beta_{p}^{\prime }{\;}^{2}}\right)}^{1/2}\\ \exp\;(-\frac{\mu_{p}}{1+\beta_{p}^{\prime 2}}\{\left(1+\beta_{p}^{\prime 2}-\beta_{p}^{2}\right)\xi_{p}^{2}-2\beta_{p}\xi_{p}\eta_{p}\\ +\left[1+\beta_{p}^{\prime }\left(\beta_{p}^{\prime }+\beta_{p}\right)\right]\eta_{p}^{2})+\left(1+\beta_{p}^{\prime 2}\right)\zeta_{p}^{2}\})\\ \beta_{p}=\dot{\gamma }b_{0}^{2}/6D_{0}\lambda_{p}\\ \beta_{p}^{\prime }=\beta_{p}\left(1+v_{p}\varphi_{p}/f\right). \end{array}$$ (13)

The index *p* runs from 1 to *Z.* The value 0 is excluded. Without internal viscosity, *φ_p_* = 0, one has *β*′*_p_ = β_p_* and one obtains the conventional distribution function. Note that eq (13) and the distribution function eq (6.1) in Ref. [1] refer to different flow fields: $\dot{\gamma }\left(y,o,o\right)$ in the former and *γ*(*z*, o, o) in the latter case.

Equation (13) explicitely contains the diagonal terms λ*_p_*, *μ_p_* and *v_p_.* In the averages (dyadic formulation)

$$\langle r^{T}Ar\rangle =b_{0}^{2}\langle u^{T}M\;u\rangle =b_{0}^{2}\sum_{p=1}^{Z}\mu_{p}\langle u_{p}u_{p}\rangle$$ (14)

which appear in the intrinsic stress and optical tensor the coefficients *μ_p_* drop completely because the averages ⟨***u****_p_****u****_p_*⟩ are proportional to 1/*μ_p_.* The coefficients *v_p_*, however, remain in *β*′*_p_* as long as *φ* ≠ 0. As already mentioned they are equal to unity (new theory) while formerly they were approximated by (λ_Z_/λ*_R_*)*_p_* = λ*_p_*(h*)/λ*_p_*(h* = 0) ≠ 1 (old theory).

In the oscillating flow field the gradient is a function of time

$$\dot{\gamma }=\gamma_{0}e^{i\omega t}.$$ (15)

The amplitude ${\dot{\gamma }}_{0}$ is so small that the molecule remains practically undeformed so that the zero gradient eigenvalues *λ_p_* and diagonal elements *v_p_* calculated for the coil at rest, are applicable.

## 4. The Intrinsic Stress Tensor

In dyadic formulation the intrinsic stress tensor reads

$$\left[\sigma \right]=\underset{c\to 0}{\lim}\frac{\sigma -\sigma_{s}}{c}=\frac{N}{M}\langle r{\;}^{T}F\rangle .$$ (14)

Here *N* is Avogadro number, *M* is molecular weight, and ***F*** is the vector of forces exerted by the beads of the necklace model on the flowing liquid. One has in the space of normal coordinates

$$F={\left(1+N\phi /f\right)}^{-1}Q^{-1T}\left[(3kT/b_{0})\;M\;u+b_{0}\phi \left(v-\Omega \times u\right)+\left(kT/b_{0}\right)\nabla \;\ln\;\psi \right]$$ (15)

which yields

$$\begin{array}{l} \left[\sigma \right]=\frac{N}{M}\left[3kT\langle u{\;}^{T}M\;u\rangle +b_{0}^{2}\langle u{\;}^{T}\phi \left(v-\Omega \times u\right)\rangle +kT\langle u{\;}^{T}\nabla \;\ln\;\psi \rangle \right].\\ {\left(1+N\phi /f\right)}^{-1} \end{array}$$ (16)

The type of laminar flow shows up in ***v*** and **Ω**.

The bilinear coordinate averages in eq (16) can be derived from the diffusion equation, eq (11), by multiplication by $\xi_{p}^{2}$, *ξ_p_η_p_*, ⋯ and integration over the whole space.

## 5. Flow With Transverse Gradient

In the case of laminar flow with transverse gradient $v+\dot{\gamma }\left(y,o,o\right)$, one obtains the set of linear differential equations

$$\begin{array}{r} \langle \xi_{p}{\;}^{2}\rangle -v_{p}/3\lambda_{p}=-2\tau_{p}\dot{\gamma }(1+v_{p}\varphi_{p}/2f)\langle \xi_{p}\eta_{p}\rangle =-\tau_{p}^{\prime }\;d\langle \xi_{p}{\;}^{2}\rangle /dt\;\\ \langle \xi_{p}\eta_{p}\rangle -\tau_{p}\gamma (v_{p}\varphi_{p}/2f)\langle \xi_{p}^{2}\rangle -\tau_{p}\dot{\gamma }(1+v_{p}\varphi_{p}/2f)\langle \eta_{p}^{2}\rangle =-\tau_{p}^{{\;}^{\prime }}\;d\langle \xi_{p}\eta_{p}\rangle /dt\\ \langle \eta_{p}^{2}\rangle -v_{p}/3\lambda_{p}+\tau_{p}\dot{\gamma }(v_{p}\varphi_{p}/f)\langle \xi_{p}\eta_{p}\rangle =-\tau_{p}^{\prime }\;d\langle \eta_{p}^{2}\rangle /dt\\ \langle \eta_{p}\zeta_{p}\rangle +\tau_{p}\dot{\gamma }\langle v_{p}\varphi_{p}/2f\rangle \langle \zeta_{p}\xi_{p}\rangle =-\tau_{p}^{\prime }\;d\langle \eta_{p}\zeta_{p}\rangle /dt\\ \langle \zeta_{p}{\;}^{2}\rangle -v_{p}/3\lambda_{p}=-\tau_{p}^{{\;}^{\prime }}\;d\langle \zeta_{p}{\;}^{2}\rangle /dt\;\\ \langle \zeta_{p}\xi_{p}\rangle +\tau_{p}\dot{\gamma }(1+v_{p}\varphi_{p}/2f)\langle \eta_{p}\zeta_{p}\rangle =-\tau_{p}^{{\;}^{\prime }}d\langle \zeta_{p}\xi_{p}\rangle /dt \end{array}$$ (17)

The steady state solutions which apply after the complete decay of transient phenomena read

$$\begin{array}{l} \langle \xi_{p}^{2}\rangle =\frac{1}{3\mu_{p}}\left[1+\frac{\gamma_{0}{\;}^{2}\tau_{p}\left(\tau_{p}^{{\;}^{\prime }}+\tau_{p}\right)/2}{1+i\omega \tau_{p}^{\prime }}\left(1+\frac{e^{2i\omega t}}{1+2i\omega \tau_{p}^{\prime }}\right)\right]\\ \langle \xi_{p}\eta_{p}\rangle =\frac{1}{3\mu_{p}}\frac{\gamma_{0}\tau_{p}}{1+i\omega \tau_{p}^{\prime }}e^{i\omega t}\\ \langle \eta_{p}^{2}\rangle =\frac{1}{3\mu_{p}}\left[1+\frac{\gamma_{0}{\;}^{2}\tau_{p}\left(\tau_{p}^{{\;}^{\prime }}-\tau_{p}\right)/2}{1+i\omega \tau_{p}^{\prime }}\left(1+\frac{e^{2i\omega t}}{1+2i\omega \tau_{p}^{\prime }}\right)\right]\\ \langle \zeta_{p}^{2}\rangle =\frac{1}{3\mu_{p}}\\ \langle \eta_{p}\zeta_{p}\rangle =\langle \zeta_{p}\xi_{p}\rangle =0 \end{array}$$ (18)

The averages are cut off beyond the lowest power *y* which is needed later in zero gradient expressions for intrinsic viscosity, normal stress difference and birefringence. These averages have to be inserted in the expression for the intrinsic stress tensor.

$$\left[\sigma \right]=\left(N/M\right)\left[3kT\langle u{\;}^{T}M\;u\rangle +\left(\dot{\gamma }b_{0}^{2}/2\right)\langle u{\;}^{T}\phi \left(\eta ,\xi ,o\right)\rangle -kT1\right]\times {\left(1+N\Phi /f\right)}^{-1}$$ (19)

yielding the frequency dependence of intrinsic viscosity

$$\begin{array}{l} \left[\eta \right]={\left[\eta \right]}_{\omega }^{\prime }-i{\left[\eta \right]}_{\omega }^{\prime \prime }\\ =\underset{c\to 0}{\lim}\frac{\eta -\eta_{s}}{c\eta_{s}}\underset{c\to 0}{\lim}\frac{\sigma_{12}*-\sigma_{12}{\;}_{s}}{c\sigma_{12}{\;}_{s}}={\left[\sigma_{12}\right]}^{*}/\eta_{s}\dot{\gamma }\\ =\frac{RT}{M\eta_{s}}\sum_{P=1}^{Z}\left(\frac{3\mu_{p}}{\dot{\gamma }}\langle \xi_{p}\eta_{p}\rangle +\frac{b_{0}{\;}^{2}\varphi_{p}}{2kT}\langle \eta_{p}{\;}^{2}\rangle \right)/\left(1+\frac{v_{p}\varphi_{p}}{f}\right)\\ \frac{RT}{M\eta_{s}}\sum \tau_{p}\frac{1+i\omega \left(\tau_{p}^{\prime }-\tau_{p}\right)}{1+i\omega \tau_{p}^{\prime }}\\ \frac{RT}{M\eta_{s}}\left[I+\text{IV}-i\left(\text{II}-\text{III}\right)\right]/\omega \\ \tan\;\delta_{\eta }=\left(\text{II}-\text{III}\right)/\left(\text{II}+\text{IV}\right) \end{array}$$ (20)

**Figure 2:**
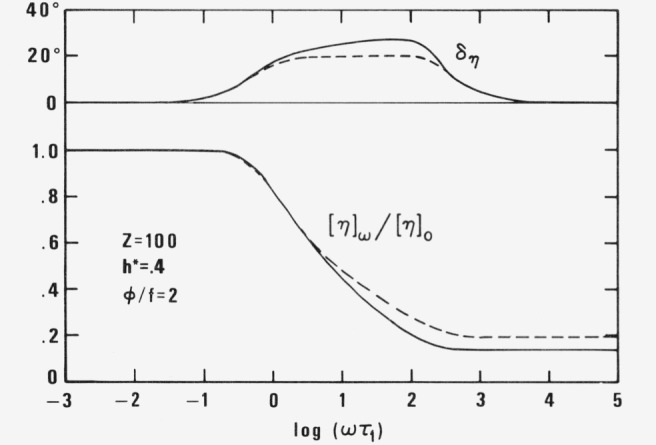
Relative intrinsic viscosity [η]_ω_/[η]_0_ oand phase angle δ_η_ for *Z* = 100, h* = .4, and φ/*f* =2 as function of ωτ_1_ according to new (full line) and old (broken line) theory.

The absolute value of intrinsic viscosity *[η]_ω_Mη_s_/RT* and the phase angle *δ_η_* as function of *ωτ*_1_ are plotted in figure 2 forc *Z* = 100, *φ/f* = 2, h* = .4 according to old and new theory.

A rather similar but not identical expression applies to intrinsic streaming birefringence

$$\begin{array}{l} {\left[\Delta n\right]}_{\omega }*={\left[\Delta n\right]}_{\omega }^{\prime }-{\left[\Delta n\right]}_{\omega }^{\prime \prime }\\ \;=\underset{c\to 0}{\lim}\frac{\Delta n*-\Delta n_{s}}{{\text{cn}}_{s}\eta_{s}\gamma }\underset{c\to 0}{\lim}\frac{\Delta n*-\Delta n_{s}}{{\text{cn}}_{s}\sigma_{12}{\;}_{s}}\\ \;=K\frac{RT}{M\eta_{s}}\sum_{P=1}^{Z}\left(\frac{2\mu_{p}}{\dot{\gamma }}\langle \xi_{p}\eta_{p}\rangle /\left(1+\frac{v_{p}\varphi_{p}}{f}\right)\right)\\ \;=\frac{4\pi }{5}{\left(\frac{n^{2}+2}{3n}\right)}^{2}\frac{N\alpha_{1}-\alpha_{2}}{M\eta_{s}}\sum \frac{\tau_{p}}{1+i\omega \tau_{p}^{\prime }}\\ \;=K\frac{RT}{M\eta_{s}}\left(I-i\text{II}\right)/\omega \\ \tan\;\delta_{\eta }=\text{II}/I. \end{array}$$ (21)

**Figure 3:**
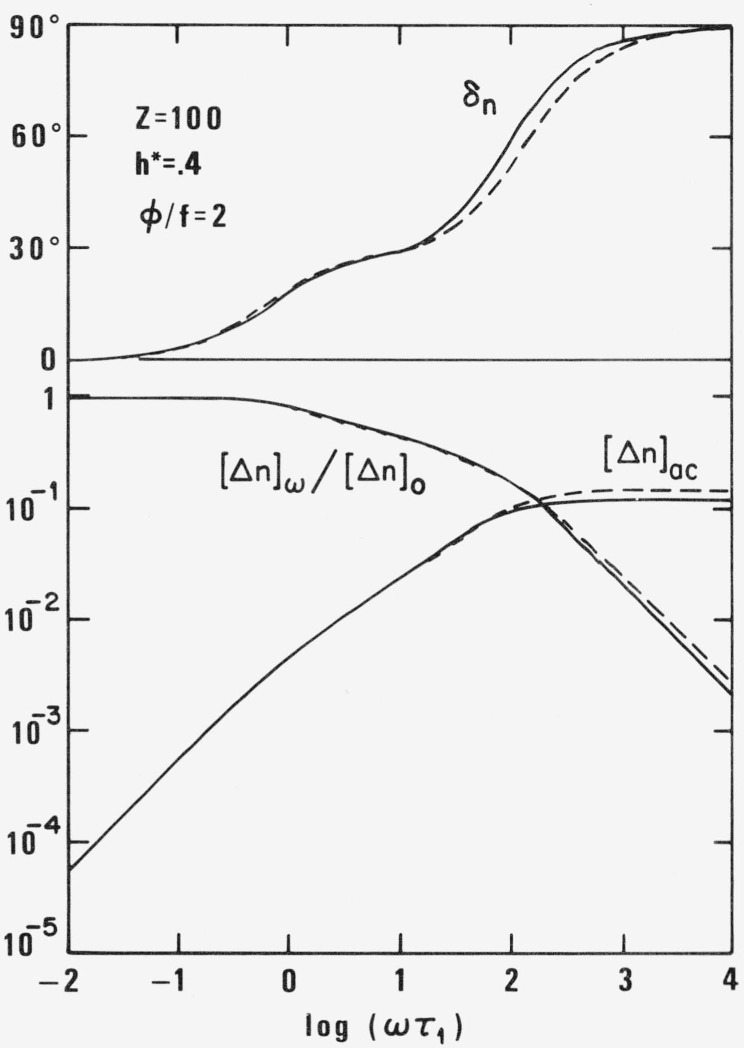
Relative intrinsic streaming birefringence [**Δ**n]_w_/[**Δ***n*]_0_ intrinsic accoustic birefringence [**Δ**n]_ac_ and phase angle δ_η_ for *Z* = 100, h* = .4, and < φ/*f* = 2 as function of ωτ_1_ according to new (full line) and old (broken line) theory.

In the case of dynamic birefringence **Δ***n* is the difference between the refractive indices in the diagonal direction in the first and second quadrant. The extinction angle *χ* = 45° as long as the gradient amplitude $\dot{\gamma }$ is small enough. The birefringence *[***Δ***n]_ω_Mη_s_/KRT* and the phase angle *δ_η_* are plotted in figure 3 for the same values of *Z*, *φ/f* and h* as in figure 2.

The meaning of the symbols in eqs (17) to (21) is as follows:

$$\begin{array}{l} \tau_{p}=b_{0}{\;}^{2}/6D_{0}\lambda_{p}\\ \tau_{p}^{\prime }=\tau_{p}\left(1+\nu_{p}\varphi_{p}/f\right)\\ \tau_{p}^{\prime }-\tau_{p}=\tau_{p}\nu_{p}\varphi_{p}/f\\ K=\frac{4\pi }{5}{\left(\frac{n^{2}+2}{3n}\right)}^{2}\left(\frac{\alpha_{1}-\alpha_{2}}{kT}\right)\\ I=\sum \omega \tau_{p}/\left(1+\omega^{2}\tau_{p}^{\prime 2}\right)\\ \text{II}=\sum \omega^{2}\tau_{p}\tau_{p}^{\prime }/\left(1+\omega^{2}\tau_{p}^{\prime 2}\right)\\ \text{III}=\sum \omega^{2}\tau_{p}\left(\tau_{p}^{\prime }-\tau_{p}\right)/\left(1+\omega^{2}\tau_{p}^{\prime 2}\right)\\ \text{IV}=\sum \omega^{3}\tau_{p}\tau_{p}^{\prime }\left(\tau_{p}^{\prime }-\tau_{p}\right)/\left(1+\omega^{2}\tau_{p}^{\prime 2}\right) \end{array}$$

The sums III and IV go to zero for vanishing internal viscosity *φ*→0. In this limiting case the intrinsic viscosity and streaming birefringence are proportional to each other. Here *R* is the gas constant, ***K*** is the rheooptical coefficient, *n* is refractive index, *δ* is the phase angle between the flow gradient and viscosity or birefringence, *α*_1_ and *α*_2_ are the optical polarizabilities of the link in the directions parallel and perpendicular to the link respectively.

Both definitions of intrinsic viscosity and birefringence in eqs (20) and (21) tend to make the intrinsic values as much as possible independent of the specific properties of the solvent, i.e., of *η_s_* and *n_s_* which influence the orientational forces, rigidity and optical anistropy of the dissolved macromolecule [52].

It is important to note that as a consequence of the new terms III and IV, i.e., 〈*η_p_*^2^〉, caused by the introduction of internal viscosity the expressions for intrinsic viscosity and streaming birefringence, eq. (20) and (21), respectively, are not proportional to each other. Hence the rheooptical law does not apply to such a model. In particular [Δ*n*]_ω_ cannot be written as *K*[*η*]*ω ~* [*σ*]_12_.

The proportionality, however, still holds in the first Newtonian region, *ω*→0, where all the terms but *I/ω* go to zero. This means that the intrinsic viscosity [*η*] and the Maxwell constant [Δ*n*] and hence the stress and the optical tensor are proportional to each other for $\dot{\gamma }\to 0$ and *ω* → 0. This is the case in most applications of flow stress mapping by means of streaming birefringence of dilute polymer solutions.

With increasing frequency and finite internal viscosity, [Δ*n*]*ω* goes to zero while [*η*]*_ω_* tends to finite value. The rheooptical law breaks down completely. In this second Newtonian range the intrinsic viscosity is independent of frequency. One derives from eq (20)

$${\left[\eta \right]}_{\infty }=\frac{RT}{M\eta_{s}}\sum_{p=1}^{z}\frac{\tau_{p}}{1+f/\nu_{p}\varphi_{p}}.$$ (23)

**Figure 4:**
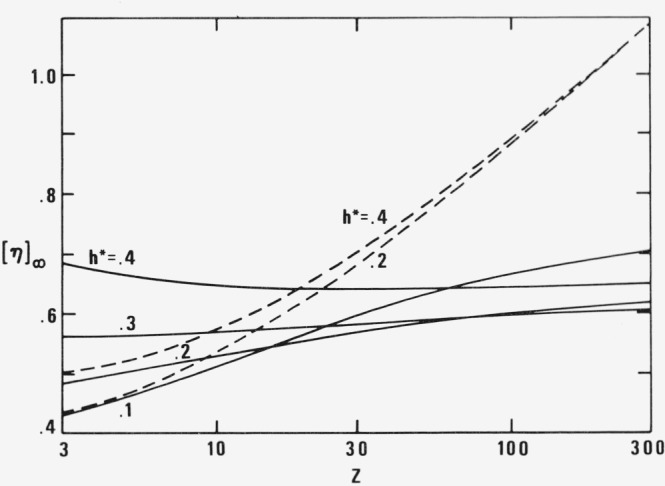
Second Newtonian intrinsic viscosity [η]_∞_ as function of number of links *Z* and hydrodynamic interaction *h* as parameter for φ/*f* = 2. New (full line) and old (broken line) theory.

The limiting values [*η*]_∞_M*η*_s_/RT for Z between 3 and 300 and h* = .1, .2, .3, .4 are plotted in figure 4 for the new (full line) and old (broken line) theory. One sees that the absolute values for *v_p_* = 1 and $v_{p}^{\left(1\right)}=\lambda_{pZ}/\lambda_{pR}$ differ by less than a factor of 2. But their dependence on *Z*, i.e., on molecular weight, is just the opposite for h* = .4. In the incorrect formulation of the old theory [*η*]_∞_ slightly increases with *Z* while in the correct formulation of the new theory it decreases with *Z*. Such a dependence on *Z* is much more in agreement with experimental data on polystyrene in Aroclor [44]. The second Newtonian viscosity [*η*]_∞_ is 14.3 cm^3^/g if one goes with ***M*** from 20,000 to 860,000. The old theory yields a steady increase of [*η*]_∞_ with ***M*** in sharp disagreement with these data.

**Figure 5:**
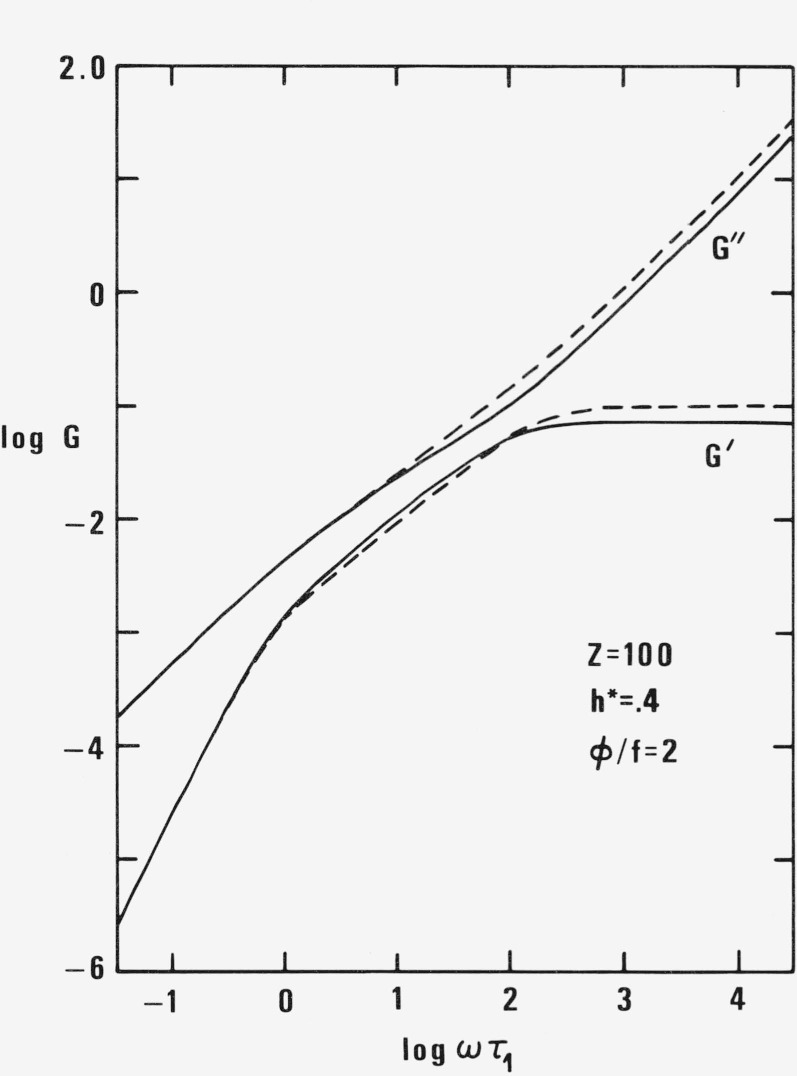
Intrinsic shear moduli [*G*]′ and [*G*]″ for *Z* = 100, h* = .4, and φ/f = 2 as functions of ωτ_1_ according to new (full line) and old (broken line) theory.

The often used real and imaginary part of intrinsic shear modulus

$$\begin{array}{l} \left[G\right]*=\left[G\right]\prime +i{\left[G\right]}^{\prime \prime }=i\omega \eta_{s}{\left[\eta \right]}_{\omega }^{*}\\ \;=\frac{1}{\dot{\gamma }}\left[\sigma_{12}\right]*=\underset{c\to 0}{\lim}\frac{\sigma_{12}^{*}-\alpha_{12s}}{c\dot{\gamma }} \end{array}$$ (24)

are plotted in figure 5. The difference between the old and new theory is relatively small for *Z* = 100 but would be larger for *Z* = 300.

The consequence of the non-vanishing second Newtonian viscosity, [*η*]_∞_ ≠ 0, is the linear increase of [*G*]′ with *ωτ*_1_ in the assymptotic high frequency region. Such a behavior is in perfect agreement with experimental data on polystyrene in highly viscous Aroclor [44–51]. These data together with the zero gradient intrinsic orientation data of streaming birefringence of polystyrene in solvents of increasing viscosity [53] constitute the main support of the theory.

The intrinsic first normal stress difference turns out to be [σ_11_–σ_22_]*

$$\begin{array}{l} =\frac{RT}{M}\overset{Z}{\underset{p=1}{\sum }}(3\mu_{p}\langle \xi_{p}^{2}-\eta_{p}^{2}\rangle +\frac{\dot{\gamma }b_{0}^{2}\varphi_{p}}{kT}\langle \xi_{p}\eta_{p}\rangle )/(1+v_{p}\varphi_{p}/f)\\ =\frac{RT}{M}\gamma_{0}^{2}\overset{Z}{\underset{p=1}{\sum }}\frac{\tau_{p}^{2}}{1+i\omega \tau_{p}^{\prime }}\cdot [1+(\frac{1}{1+i\omega \tau_{p}^{\prime }}+\frac{\tau_{p}^{\prime }-\tau_{p}}{\tau_{p}})e^{2i\omega t}]\\ =A*e^{2i\omega t}+B* \end{array}$$ (25)

while the second normal stress difference vanishes in all isolated necklace models with ideal elastic links independent of internal viscosity. In applying eq (25) one must not forget that the phase angle δ_1_ of A* is dependent only on the factor of exp (2*iωt*) and that the angle δ_2_ only reduces the constant vertical displacement to *B* cos δ_2_ but does not yield any phase shift.

**Figure 6:**
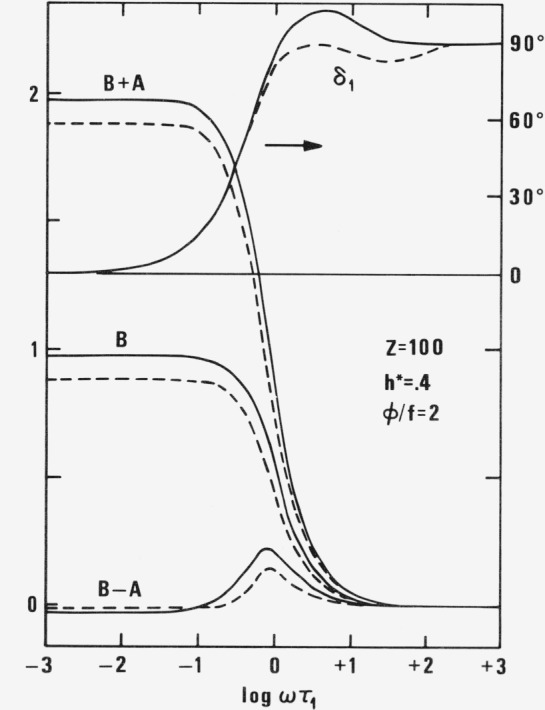
The coefficient *B*, the sum *B + A*, the difference *B — A* of the relative intrinsic normal stress difference [σ_11_–σ_22_]_ω_/[σ_11_–σ_22_]_0_ = *A e*1^2iωτ−iδ1^ + *B* for *Z* = 100, h* = .4 and φ/f = 2 as functions of ωτ_1_ according to new (full line) and old (broken line) theory.

The term 1 in the parenthesis, i.e., B*, keeps the first normal stress difference positive during the whole period up to very high frequencies. The oscillation is taking place with twice the frequency of the flow field. All these effects are very much the same as in the case of no internal viscosity. The difference is mainly in the replacement of ***τ****_p_* by ***τ′****_p_* and the term proportional to ***τ′****_p_ —* ***τ****_p_.* The amplitude *A* of the oscillating term

$$\begin{array}{l} A*=Ae^{-i\delta_{1}}\\ =\frac{RT}{M}{\dot{\gamma }}_{p}{\;}^{2}\overset{Z}{\underset{p=1}{\sum }}\frac{\tau_{p}}{1+i\omega \tau_{p}^{\prime }}(\frac{1}{1+2i\omega \tau_{p}^{\prime }}+\frac{\tau_{p}^{\prime }-\tau_{p}}{\tau_{p}})[1+2\omega^{2}\tau_{p}^{\prime }(2\tau_{p}^{\prime }-\tau_{p})]\\ =\frac{RT}{M}{\dot{\gamma }}_{0}{\;}^{2}\overset{Z}{\underset{p=1}{\sum }}\tau_{p}{\;}^{2}\frac{-i\omega [\tau_{p}^{\prime }+2\tau_{p}+4\omega^{2}\tau_{p}^{\prime }{\;}^{2}(\tau_{p}^{\prime }-\tau_{p})]}{\left(1+\omega^{2}\tau_{p}^{\prime }{\;}^{2})(1+4\omega^{2}\tau_{p}^{\prime }{\;}^{2}\right)} \end{array}$$ (26)

is proportional to the square of the amplitude of the oscillating gradient as in the case of no internal viscosity. The value (*A/γ_0_^2^)(M/RT*), the phase angle δ_l_ and the frequency independent displacement

$$\left(B\cdot \cos\;\delta_{2}/{\dot{\gamma }}_{0}{\;}^{2}\right)\left(M/RT\right)=\sum_{p=1}^{Z}\frac{\tau_{p}{\;}^{2}}{1+\omega^{2}\tau_{p}^{\prime }{\;}^{2}}$$ (27)

are plotted in figure 6 as functions of *ωτ*_1_ for *Z* = 100 and h* = .4. Both quantities *A* and *B* are going to zero with increasing frequency. In contrast to intrinsic viscosity the limiting first normal stress difference, at *ω* → ∞, does not become finite by introduction of internal viscosity although it goes to zero more slowly, as *ω*^−1^ instead of as *ω*^−3^.

## 6. Flow With Longitudinal Gradient

In jet flow, $v=\dot{\gamma }(-x/2,\;-y/2,z)$ the intrinsic stress tensor

$$\begin{array}{l} {}[\sigma ]=\frac{N_{A}}{M}[3kT\langle u\;M\;u\rangle \\ \;+(\dot{\gamma }b_{0}{\;}^{2}/2)\langle u\phi (-\xi ,-\eta ,+2\eta )\rangle +kT1]\\ \;\times {\left(1+N\;\phi /f\right)}^{-1} \end{array}$$ (28)

is independent of coil rotation in flow because the flow field has no rotational component, Ω = 0. The bilinear coordinate averages are derivable from the set of differential equations

$$\begin{array}{r} (1+\dot{\gamma }\tau_{p})\langle \xi_{p}{\;}^{2}\rangle -v_{p}/3\lambda_{p}=-\tau_{p}^{{\;}^{\prime }}\;d\langle \xi_{p}{\;}^{2}\rangle /\;dt\;\\ (1+\gamma \tau_{p})\langle \xi_{p}\eta_{p}\rangle =-\tau_{p}^{{\;}^{\prime }}\;d\langle \xi_{p}\eta_{p}\rangle /\;dt\\ (1+\dot{\gamma }\tau_{p})\langle \eta_{p}{\;}^{2}\rangle -v_{p}/3\lambda_{p}=-\tau_{p}^{{\;}^{\prime }}\;d\langle \eta_{p}{\;}^{2}\rangle /\;dt\\ (1-\gamma \tau_{p}/2)\langle \eta_{p}\zeta_{p}\rangle =-\tau_{p}^{{\;}^{\prime }}\;d\langle \eta_{p}\zeta_{p}\rangle /dt\\ (1-\dot{\gamma }\tau_{p})\langle \zeta_{p}{\;}^{2}\rangle -v_{p}/3\lambda_{p}=-\tau_{p}^{{\;}^{\prime }}\;d\langle \zeta_{p}{\;}^{2}\rangle /dt\;\\ (1-\gamma \tau_{p}/2)\langle \zeta_{p}\xi_{p}\rangle =-\tau_{p}^{{\;}^{\prime }}\;d\langle \zeta_{p}\xi_{p}\rangle /dt \end{array}$$ (29)

Under consideration of the symmetry of flow field one obtains

$$\begin{array}{l} \langle \xi_{p}{\;}^{2}\rangle =\langle \eta_{p}{\;}^{2}\rangle =\frac{1}{3\mu_{p}}\exp\;\{-[t+({\dot{\gamma }}_{0}\tau_{p}/i\omega )e^{i\omega t}]/\tau_{p}^{{\;}^{\prime }}\}\\ \times \overset{t/\tau^{\prime }{\;}_{p}}{\underset{0}{\int }}\exp\;[x+({\dot{\gamma }}_{0}\tau_{p}/i\omega \tau_{p}^{{\;}^{\prime }})e^{i\omega \tau^{\prime }{\;}_{p}x}]\;dx\\ \langle \xi_{p}{\;}^{2}\rangle =\frac{1}{3\mu_{p}}\exp\;\{-[t-2({\dot{\gamma }}_{0}\tau_{p}/i\omega )e^{i\omega t}]/\tau_{p}^{{\;}^{\prime }}\}\\ \begin{array}{l} \times \overset{t/\tau^{\prime }{\;}_{p}}{\underset{0}{\int }}\exp\;[x-2({\dot{\gamma }}_{0}\tau_{p}/i\omega \tau_{p}^{{\;}^{\prime }})e^{i\omega \tau^{\prime }{\;}_{p}x}]dx\\ \langle \xi_{p}\eta_{p}\rangle =\langle \eta_{p}\xi_{p}\rangle =\langle \xi_{p}\zeta_{p}\rangle =0. \end{array} \end{array}$$ (30)

**Figure 7:**
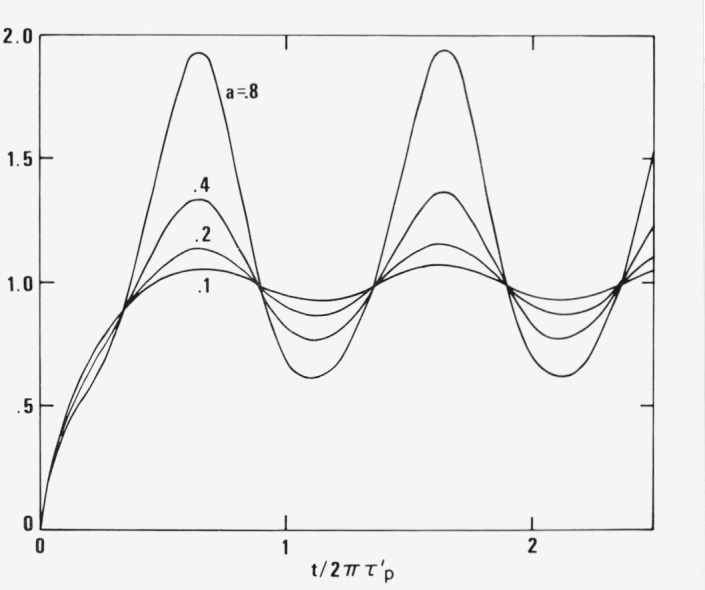
Time dependence of $\langle \xi_{p}{\;}^{2}\rangle =\langle \eta_{p}{\;}^{2}\rangle$ according to eq (30) plotted versus t/2πτ′_p_ for the special case $\varphi \tau_{p}^{{\;}^{\prime }}=1$ and a = $a={\dot{\gamma }}_{0}\tau_{p}/\omega \tau_{p}^{{\;}^{\prime }}$ showing the short duration of transient effects and the rapid approach to the asymptotic periodic solution. The stabilization is slower for low ωτ'_p_.

These averages still contain the transient which, in the general case, cannot be easily separated from the stationary solution reached after the transient has tapered off (fig. 7).

The separation can be performed if ${\dot{\gamma }}_{0}\tau_{p}/\omega \tau_{p}^{{\;}^{\prime }}$ is so small that one can replace the exponential function by its linear expansion. In such a case one obtains for the stationary solution

$$\begin{array}{l} \langle \xi_{p}{\;}^{2}\rangle =\frac{1}{3\mu_{p}}\left(1-{\dot{\gamma }}_{0}\tau_{p}\frac{e^{i\omega t}}{1+i\omega \tau_{p}^{{\;}^{\prime }}}\right)\\ \langle \zeta_{p}{\;}^{2}\rangle =\frac{1}{3\mu_{p}}\left(1+2{\dot{\gamma }}_{0}\tau_{p}\frac{e^{i\omega t}}{1+i\omega \tau_{p}^{{\;}^{\prime }}}\right) \end{array}$$ (31)

The intrinsic Trouton viscosity turns out to be

$$\begin{array}{l} {\left[\eta \right]}_{l\omega }^{*}\\ =[\sigma_{33}-\sigma_{11}]*/\gamma \eta_{s}\\ =\frac{RT}{M\eta_{s}}\overset{Z}{\underset{p=1}{\sum }}3\mu_{p}[\langle \zeta_{p}{\;}^{2}-\xi_{p}{\;}^{2}\rangle /\dot{\gamma }+(\tau_{p}v_{p}\varphi_{p}/f)\langle 2\zeta_{p}{\;}^{2}-\xi_{p}{\;}^{2}\rangle ]\\ =\frac{3RT}{M\eta_{s}}\overset{Z}{\underset{p=1}{\sum }}(\frac{\tau_{p}(1-\omega \tau_{p}^{{\;}^{\prime }}}{1+\omega^{2}\tau_{p}^{{\;}^{\prime 2}}}+\frac{\tau_{p}^{{\;}^{\prime }}-\tau_{p}}{3})\\ \frac{3RT}{M\eta_{s}}(I/\omega +\overset{Z}{\underset{p=1}{\sum }}(\tau_{p}^{{\;}^{\prime }}-\tau_{p})/3-i\text{II}/\omega ). \end{array}$$ (32)

**Figure 8:**
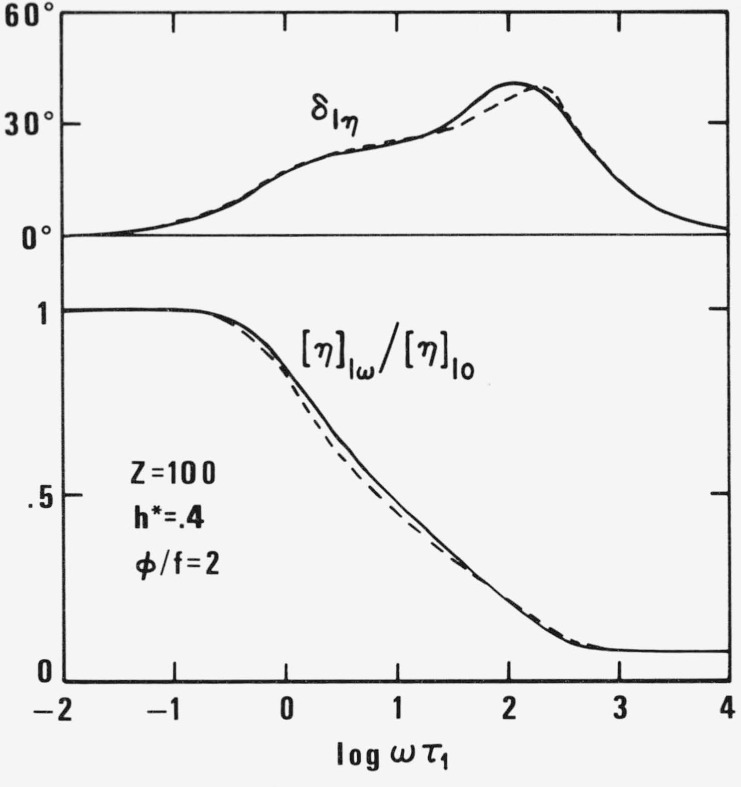
Relative intrinsic Trouton viscosity [η]_lω_[η]_l__0_ and the phase angle δ_lη_ for *Z* = 100, h* = .4, and φ/*f* = 2 as function of ωτ_1_ according to new (full line) and old (broken line) theory.

The additional term $\sum (\tau_{p}^{{\;}^{\prime }}-\tau_{p})/3$ is independent of *ω* and hence represents the Trouton viscosity in the second Newtonian range where I/*ω* and II/*ω* disappear. As in the case of conventional intrinsic viscosity, eq (20), the finite value at co *ω*→∞ is a consequence of internal viscosity, i.e., of partial coil rigidity. The frequency dependence of [*η*]*_ω_* and phase angle *δ_lη_* are plotted in figure 8 for *Z* = 100, h* = .4, and *φ*/*f*=2.

The intrinsic birefringence reads

$${\left[\Delta n\right]}_{l\omega }^{*}=K\frac{RT}{M\eta_{s}\dot{\gamma }}\sum_{p=1}^{Z}3\mu_{p}\langle \zeta_{p}{\;}^{2}-\xi_{p}{\;}^{2}\rangle$$ (33)

which for small amplitude reduces to

$$\begin{array}{l} {\left[\Delta n\right]}_{l\omega }^{*}=\frac{3KRT}{M\eta_{s}}\cdot \overset{Z}{\underset{p=1}{\sum }}\tau_{p}/(1+i\omega \tau_{p}^{{\;}^{\prime }})=\frac{3KRT}{M\eta_{s\omega }}\cdot (\text{I}-i\text{II})\\ \tan\;\delta_{ln}=\text{II}/\text{I} \end{array}$$ (34)

This expression differs from the streaming birefringence in an oscillating flow field eq (21), only in the factor 3. The nonproportionality between viscosity and birefringence is again the consequency of the additional internal viscosity term in eq (33).

Acoustic birefringence is in many respects closely related to birefringence in an oscillating jet flow. The main difference is the absence of lateral contraction as represented by $-\dot{\gamma }x/2$ and $-\dot{\gamma }x/2$. In contrast to jet flow with constant specific volume (incompressible liquid) the volume element subjected to an acoustic wave is periodically compressed and expanded. That means a constant 〈ξ*_p_*^2^〉 yielding in eq (34) the replacement of the factor 3 by 2.

One has

$$\begin{array}{l} {\left[\Delta n\right]}_{ac}^{*}=\underset{c\to 0}{\lim}\frac{\text{Δn}}{nc{\text{I}}_{ac}^{1/2}}\\ =\frac{N}{M}K_{ac}\frac{\omega \tau }{{\left(1+\omega^{2}\tau^{2}\right)}^{1/2}}\sin[\omega (t-z_{0}/c_{a})-\delta_{ac}]\\ =\frac{N}{M}\cdot K_{ac}\cdot \overset{Z}{\underset{p=1}{\sum }}\frac{-i\omega \tau_{p}}{1+i\omega \tau_{p}^{{\;}^{\prime }}}e^{i\omega (t-z_{0}/c_{a})}\\ =\frac{N}{M}K_{ac}(\text{I}-i\text{II})e^{i\omega (t-z_{0}/c_{a})}\\ \tan\;\delta_{ac}=\text{II}/\text{I}\\ K_{ac}=\frac{4\pi }{5}{\left(\frac{n^{2}+2}{3n}\right)}^{2}{\left(\frac{2}{\rho_{c^{3}ac}}\right)}^{1/2}(\alpha_{1}-\alpha_{2})\\ {\text{I}}_{ac}=\rho B^{2}c_{ac}/2. \end{array}$$ (35)

Here *z*_0_ is the location of the center of hydrodynamic resistance of the macromolecule, B is amplitude, I*_ac_* is intensity and *c_ac_* is propagation velocity of acoustic wave, and *ρ* is the density of solution. The frequency dependence of acoustic birefringence and phase angle is exactly the same as in the case of oscillating jet flow.

The difference between acoustic birefringence according to the correct new and the incorrect old values ***v****_p_* can be seen in figure 3 where [Δ*n*]*_ac_* is plotted versus ωτ_1_ for *Z* = 100, h* = .4 and *φ*/*f* = 2. The phase angle *δ_ac_* is identical with *δ_n_* and *δ_ln_*.

## 7. Conclusions

The paper presents the calculation of most of the intrinsic rheological and rheooptical effects of linear homopolymers in an oscillating flow field which may be explored experimentally. In the case of rheooptical effects only the intrinsic birefringence of the polymer is included. The form-birefringence is completely neglected. The same applies to the influence of large side groups which may effect independently the optical anisotropy of the segment and its frequency dependence.

The introduction of the appropriate ***v****_p_* = 1 values instead of the old values ***v****_p_* = **λ***_pZ_*/**λ***_pR_* does not change drastically the effects depending on internal viscosity. As a rule the ratio between the new and old values of intrinsic viscosity, birefringence, and first normal stress difference is less than 2, at least in the range of *Z* between 3 and 300. With higher *Z* the differences increase as a linear function of log *Z*.

As already mentioned, the smallness of the difference is a consequence of the peculiar dependence of old *v^p^*^(1)^ on *p:* much larger than 1 at small *p* and smaller than 1 at high *p.* Hence the larger contributions in the former part of the sums are partially compensated by the smaller contributions in the latter part.

The most important changes occur in the second Newtonian intrinsic viscosity which is the most conspicuous consequence of internal viscosity. Here the dependence of [η]_∞_ on *Z* is much less according to the new theory than it was in the case of the old theory.

No calculation of the gradient effects was attempted because one knows that in a flow with a finite velocity gradient the randomly coiled macromolecule is deformed with a consequent change of all interbead distances. This yields a change of interaction tensor **H** which leads to a modification of all eigenvalues **λ***_p_.* The rest values **λ***_p_* used in this paper are only applicable to effects where an extrapolation to zero gradient is straightforward. This is the case with dynamic effects where one uses very small gradients and concentrates on the frequency dependence of the effects measured. The situation, however, is basically different in the non-linear range of gradient dependence of excess stress and optical tensor.

## Figures and Tables

**Table 1:** Eigenvalues λ*_p_* for *Z* = 100 and h* = 0 (Rouse), .1, .2, .3, .4 (Zimm)

*p*	H^*^ = 0	.1	.2	.3	.4	*P*	h^*^ = 0	.1	.2	.3	.4

1	0.000967	0.002170	0.003345	0.004506	0.005643	51	2.03111	1.7822	1.5334	1.2846	1.0359
2	.003869	.007318	.010718	.014084	.017417	52	2.09329	1.8320	1.5707	1.3094	1.0483
3	.008701	.014650	.020541	.026383	.032173	53	2.15538	1.8815	1.6077	1.3339	1.0602
4	.015460	.023986	.032447	.040848	.049177	54	2.21731	1.9308	1.6443	1.3579	1.0716
5	.024139	.035175	.046144	.057043	.067854	55	2.27904	1.9799	1.6807	1.3816	1.0826
6	.034730	.048140	.061482	.074746	.087907	56	2.34050	2.0286	1.7167	1.4049	1.0932
7	.047220	.062802	.078315	.093745	.10906	57	2.40153	2.0770	1.7523	1.4277	1.1033
8	.061602	.079111	.096552	.11391	.131113	58	2.46236	2.1249	1.7875	1.4502	1.1130
9	.077859	.097010	.11610	. 13509	.15395	59	2.52265	2.1725	1.8223	1.4722	1.1223
10	.095974	.11646	.13688	.15721	.17740	60	2.58244	2.2195	1.8566	1.4938	1.1311
11	.115932	.13742	.15884	.18017	.20135	61	2.64166	2.2661	1.8905	1.5150	1.1396
12	.137713	.15984	.18192	.20390	.22571	62	2.70026	2.3120	1.9238	1.5357	1.1477
13	.161295	.18370	.20605	.22831	.25040	63	2.75819	2.3574	1.9566	1.5559	1.1554
14	.186656	.20895	.23120	.25335	.27533	64	2.81538	2.4021	1.9889	1.5757	1.1627
15	.213771	.23556	.25730	.27896	.30044	65	2.87178	2.4462	2.0206	1.5950	1.1697
16	.242614	.26350	.28434	.30509	.32566	66	2.92734	2.4895	2.0517	1.6139	1.1763
17	.273158	.29272	.31224	.33169	.35095	67	2.98200	2.5321	2.0821	1.6323	1.1826
18	.305372	.32320	.34099	.35870	.37623	68	3.03571	2.5738	2.1120	1.6502	1.1885
19	.339225	.35490	.37053	.38610	.40148	69	3.08842	2.6148	2.1411	1.6676	1.1941
20	.374681	.38778	.40084	.41383	.42664	70	3.14007	2.6548	2.1696	1.6845	1.1995
21	.411719	.42180	.43186	.44186	.45168	71	3.19063	2.6940	I 2.1974	1.7009	1.2045
22	.450288	.45694	.46358	.47015	.47655	72	3.24003	2.7323	2.2245	1.7168	1.2092
23	.490356	.49316	.49594	.49867	.50123	73	3.28823	2.7695	2.2508	1.7322	1.2137
24	.531886	.53041	.52892	.52738	.52568	74	3.33518	2.8058	2.2764	1.7471	1.2179
25	.574835	.56867	.56248	.55626	.54987	75	3.38085	2.8410	2.3012	1.7614	1.2218
26	.619163	.60788	.59659	.58527	.57378	76	3.42518	2.8752	2.3252	1.7753	1.2255
27	.664827	.64802	.63122	.61438	.59738	77	3.46812	2.9083	2.3484	1.7886	1.2290
28	.711782	.68905	.66632	.64356	.62065	78	3.50965	2.9402	2.3708	1.8014	1.2322
29	.759984	.73093	.70187	.67279	.64357	79	3.54972	2.9710	2.3924	1.8137	1.2352
30	.809386	.77361	.73784	.70205	.66613	80	3.58829	3.0007	2.4131	1.8255	1.2380
31	.859939	.81706	.77419	.73130	.68829	81	3.62532	3.0291	2.4329	1.8367	1.2406
32	.911596	.86124	.81089	.76053	.71005	82	3.66078	3.0563	2.4518	1.8474	1.2430
33	.964305	.90610	.84790	.78970	.73140	83	3.69464	3.0823	2.4699	1.8575	1.2453
34	1.01802	.95160	.88521	.81881	.75231	84	3.72685	3.1069	2.4870	1.8672	1.2474
35	1.07268	.99771	.92277	.84782	.77279	85	3.75737	3.1303	2.5033	1.8762	1.2493
36	1.12824	1.0444	.96055	.87672	.79281	86	3.78624	3.1524	2.5186	1.8848	1.2510
37	1.18464	1.0916	.99852	.90548	.81238	87	3.81335	3.1731	2.5329	1.8928	1.2526
38	1.24183	1.1392	1.0367	.93410	.83148	88	3.83871	3.1925	2.5464	1.9002	1.2541
39	1.29975	1.1873	1.0749	.96254	.85011	89	3.86229	3.2105	2.5588	1.9071	1.2554
40	1.35835	1.2358	1.1133	.99079	.86826	90	3.88407	3.2272	2.5703	1.9134	1.2566
41	1.41758	1.2846	1.1517	1.0188	.88592	91	3.90403	3.2424	2.5808	1.9192	1.2577
42	1.47736	1.3338	1.1902	1.0467	.90311	92	3.92215	3.2563	2.5904	1.9245	1.2586
43	1.53758	1.3832	1.2287	1.0742	.91980	93	3.93840	3.2687	2.5989	1.9292	1.2595
44	1.59839	1.4328	1.2671	1.1016	.93601	94	3.95278	3.2796	1 2.6065	1.9333	1.2602
45	1.65952	1.4825	1.3056	1.1286	.95173	95	3.96527	3.2892	2.6130	1.9369	1.2608
46	1.72098	1.5324	1.3439	1.1554	.96696	96	3.97586	3.2972	2.6186	1.9400	1.2614
47	1.78270	1.5824	1.3821	1.1819	.98171	97	3.98454	3.3038	2.6232	1.9425	1.2618
48	1.84464	1.6324	1.4202	1.2021	.99597	98	3.99130	3.3090	2.6267	1.9444	1.2621
49	1.90673	1.6824	1.4581	1.2339	1.0098	99	3.99613	3.3127	2.6292	1.9458	1.2624
50	1.96890	1.7324	1.4959	1.2594	1.0231	100	3.99903	3.3149	2.6308	1.9466	1.2625

## Footnotes

1 Figures in brackets indicate the literature references at the end of this paper.
